# Prediabetes is associated with the modulation of antigen-specific Th1/Tc1 and Th17/Tc17 responses in latent *Mycobacterium tuberculosis* infection

**DOI:** 10.1371/journal.pone.0178000

**Published:** 2017-05-30

**Authors:** Nathella Pavan Kumar, Kadar Moideen, Chandrakumar Dolla, Paul Kumaran, Subash Babu

**Affiliations:** 1 National Institutes of Health—NIRT—International Center for Excellence in Research, Chennai, India; 2 National Institute for Research in Tuberculosis, Chennai, India; Universita degli Studi di Palermo, ITALY

## Abstract

Type 2 diabetes mellitus (DM) is associated with the down modulation of Th1, Th2 and Th17 responses in latent *Mycobacterium tuberculosis* infection but the role of prediabetes (PDM) in this setting is not well understood. To examine the role of CD4^+^ and CD8^+^ T cell cytokines in latent tuberculosis (LTB) with coincident PDM, we studied the baseline, mycobacterial, control antigen and mitogen–stimulated T cell cytokine responses in LTB individuals with (LTB-PDM; n = 20) or without (LTB-NDM; n = 20) concomitant prediabetes. LTB-PDM is characterized by diminished frequencies of mono–and dual–functional CD4^+^ Th1 and Th17 cells and mono-functional Th2 cells at baseline and/or following mycobacterial—antigen stimulation in comparison to LTB-NDM. LTB-PDM is also characterized by diminished frequencies of mono–functional CD8^+^ Tc1, Tc2 and Tc17 cells at baseline and/or following mycobacterial–antigen stimulation in comparison to LTB-NDM. LTB-PDM is therefore characterized by diminished frequencies of antigen–specific Th1/Tc1 and Th17/Tc17 cells, indicating that PDM is associated with alterations of the immune response in latent TB associated with compromised CD4^+^ and CD8^+^ T cell function.

## Introduction

Pre-diabetes (PDM) or intermediate hyperglycemia is a high risk state for diabetes that is characterized by levels of glucose above the normal thresholds but falling below the levels of overt diabetes [1]. The prevalence of PDM is on the increase globally, and it is estimated that over 470 million people will have PDM by 2030 [2]. The two main physiological abnormalities in PDM are insulin resistance and pancreatic beta-cell dysfunction, and these changes manifest before the occurrence of glucose levels abnormalities [1,3]. Previous studies have shown an important association between PDM and early forms of diabetic complications including nephropathy, small fiber neuropathy, retinopathy, and macrovascular disease [1,3]. It has been estimated that approximately 5–10% of individuals with PDM become diabetic every year depending on the population and geographical location [4,5].

While the role of Type 2 diabetes mellitus (DM) as an important risk factor for active pulmonary TB has been widely explored recently [6], very little is known about the role of PDM in active or latent TB. PDM has been shown recently to be associated with an increased risk of latent TB infection [7], to be associated with pulmonary TB in individuals with respiratory symptoms [8], and to be associated with dysregulated cytokine responses in pulmonary TB [9]. In addition, the prevalence of PDM has been reported to be as high as 25% in individuals with active TB [10]. Thus, in addition to overt DM, PDM also might have an important role to play in the nexus between metabolic disorders and pulmonary TB. CD4^+^ and CD8^+^ T cells play an important role in protective immunity to TB in both animal models and human infection [11]. More specifically, CD4^+^ Th1 and Th17 cells have been shown to play a critical role in protection either in primary or memory responses as well as against a variety of different TB strains [11,12]. In contrast, CD4^+^ Th2 cells are known to be detrimental in the protective immunity to TB [13]. Furthermore, dual–and multi–functional T cells, especially those secreting IL-2 and IFNγ, have also been associated with resistance to infection in animal models [14] and in some human studies [15,16]. Similarly, CD8^+^ T cells secreting Tc1 and Tc17 cytokines are also thought to play an important role in protective immunity to TB [17].

To study the influence of PDM on CD4^+^ and CD8^+^ T cell responses in LTB, we examined baseline, antigen–induced and polyclonal induction of mono-, dual- and multi-functional cells of the Th1/Tc1, Th2/Tc2 and Th17/Tc17 subsets in LTB individuals with coincident PDM (LTB-PDM) and compared them to those without PDM (LTB-NDM). We show that those with LTB-PDM have diminished frequencies of antigen–specific Th1/Tc1 and Th17/Tc17 cells. Thus, our data reveal that prediabetes is associated with alterations in the CD4^+^ and CD8^+^ T cell response to TB antigens.

## Materials and methods

### Ethical statement

All individuals were examined as part of a natural history study approved by the Institutional Review Board of the National Institute of Research in Tuberculosis (NCT00375583), and informed written consent was obtained from all participants.

### Study population

We studied a group of 40 individuals with LTB—20 with prediabetes and 20 without. All individuals were screened as part of a natural history study protocol conducted in a rural population outside Chennai, South India. This was a cross-sectional study, nested within a larger study examining the prevalence of LTB and diabetes/prediabetes in the community. Screening for LTB and prediabetes was performed at the same time point in each individual. The inclusion criteria were age 18 to 65, willingness to give informed consent and willingness to undergo study procedures. The exclusion criteria were active TB and HIV. The individuals underwent clinical examination and study procedures and answered a questionnaire about socio-demographic and other parameters. LTB was diagnosed on the basis of being positive for both the tuberculin skin test (>12 mm) and for Quantiferon TB Gold-in-tube assay (Qiagen, Valencia, CA) with absence of pulmonary symptoms and normal chest radiographs. TST was performed by an intradermal injection of 2 TU of Serum Statens Institute tuberculin (RT23) in the inner forearm and read at 48 hours. They were not known contacts of active TB cases. PDM was diagnosed on the basis of glycated hemoglobin (HbA1c) levels, according to the American Diabetes Association criteria (HbA1c >5.7% and <6.4%). All non-DM individuals has HbA1c levels < 5.7%. HbA1c was measured by the direct enzymatic assay method (Diazyme Laboratories, Poway, CA). All the individuals were HIV negative based on the Alere Determine HIV1/2 antibody test. All individuals were anti-TB treatment naive. Biochemical parameters, including plasma glucose, urea, creatinine, aspartate amino transferase (AST) and alanine amino transferase (ALT) were obtained using standardized techniques.

### Antigens

TB antigens used were PPD (Serum Statens Institute, Denmark), ESAT-6 peptide pools and CFP-10 peptide pools (both from BEI resources, NIAID, NIH). Influenza virus (Flu) peptide pools (BEI resources, NIAID, NIH) was used as a control antigen. Each peptide pool comprised of 10 overlapping peptides (15 amino acid long with 11 overlaps) and was used at 1μg/ml of each peptide in the pool. Final concentrations were 10 μg/ml for PPD, ESAT-6, CFP-10 and Flu peptide pools. Phorbol myristoyl acetate and ionomycin (P/I), at concentrations of 12.5 ng/ml and 125 ng/ml (respectively), were used as the positive control stimuli.

### *In vitro* culture

Whole blood cell cultures in duplicates were performed to determine the intracellular levels of cytokines.. Briefly, whole blood was diluted 1:1 with RPMI-1640 medium, supplemented with penicillin/streptomycin (100 U/100 mg/ml), L-glutamine (2 mM), and HEPES (10 mM) (all from Invitrogen, Carlsbad, CA) and distributed in 12-well tissue culture plates (Corning Costar, Glendale, AZ). The cultures were then stimulated with PPD, ESAT-6, CFP-10, Flu or P/I or media alone in the presence of the co-stimulatory molecules, CD49d /CD28 at 37°C for 6 hrs. Brefeldin A (10μg/ml) was added after 2 hours. After 6 hours, centrifugation, washing and red blood cell lysis was performed. The cells were fixed using cytofix/cytoperm buffer (BD Biosciences) and cryopreserved at -80°C.

### Intracellular cytokine staining

The cells were thawed, washed and then stained with surface antibodies for 30–60 minutes. Surface antibodies used were CD3, CD4 and CD8. The cells were washed and permeabilized with BD Perm/Wash buffer (BD Biosciences, San Jose, CA) and stained with intracellular cytokines for an additional 30 min before washing and acquisition. Cytokine antibodies used were IFNγ, TNFα, IL-2, IL-17F, IL-17A, IL-4, IL-5 and IL-13. Eight-color flow cytometry was performed on a FACS Canto II flow cytometer with FACS Diva software v.6 (Becton Dickinson, Franklin Lakes, NJ). The lymphocyte gating was set by forward and side scatter and 100,000 lymphocytes events were acquired. Data were collected and analysed using Flow Jo software (TreeStar Inc, Ashland, CA). All data are depicted as frequency of CD4^+^ and CD8^+^ T cells expressing cytokine(s). Baseline values following media stimulation are depicted as baseline frequency while frequencies following stimulation with antigens are depicted as net frequencies (with baseline values subtracted).

### Statistical analysis

Data analyses were performed using GraphPad PRISM (GraphPad Software, Inc, La Jolla, CA). Geometric means (GM) were used for measurements of central tendency. Statistically significant differences between two groups were analyzed using the nonparametric two sided Mann-Whitney U test. Multiple comparisons were corrected using the Holm’s correction. P values < 0.05 were considered statistically significant.

## Results

### Study population characteristics

The baseline characteristics including demographics, clinical and biochemical features of the study population are shown in Table 1. Individuals with LTB and diabetes (LTB-DM) had higher glycated hemoglobin than individuals with LTB and no diabetes (LTB-NDM) but no significant difference in random plasma glucose, ALT, AST, urea or creatinine. The groups did not differ significantly in age, sex or body mass index.

**Table 1:** Demographics and biochemical parameters of the study population.

Study Demographics	LTB-PDM	LTB-NDM	P Value
**No. of subjects recruited**	20	20	
**Gender (M/F)**	10-Oct	19-Nov	p = 0.8977
**Median Age (Range)**	45.5 (28–65)	33.3 (19–60)	p = 0.5123
**Body Mass Index** ^**a**^	23.3 (15.2–31.2)	22.7 (13.9–32.3)	p = 0.6781
**Mantoux Skin test Positive >12mm**	15 (12–18)	15(12–18)	p = 0.8946
**Interferon gamma release assay**	Positive 5.1 (3.26–10)	Positive 4.9 (3.79–10)	p = 0.7453
**Random Blood Glucose, mg/dl**	112 (69–237)	88 (71–139)	p = 0.1699
**Glycated hemoglobin level, %**	6.2 (5.7–6.4)	5.1 (4.57–5.36)	**p<0.0001**
**AST, U/l**	23.1 (13–58)	24 (13–52)	p = 0.7771
**ALT, U/l**	22 (12–57)	20.2 (9–49)	p = 0.5361
**Urea, mg/dl**	22 (15–49)	19.5 (11–29)	p = 0.7997
**Creatinine, mg/dl**	0.89 (0.6–1.3)	0.73 (0.3–1.2)	p = 0.9071

Values represent the geometric mean or median (and range) and the p values were calculated using the Mann-Whitney U test.

### LTB-PDM is associated with decreased frequencies of antigen-induced mono–, dual–and multi–Functional CD4^+^ Th1 cells

**Fig 1:**
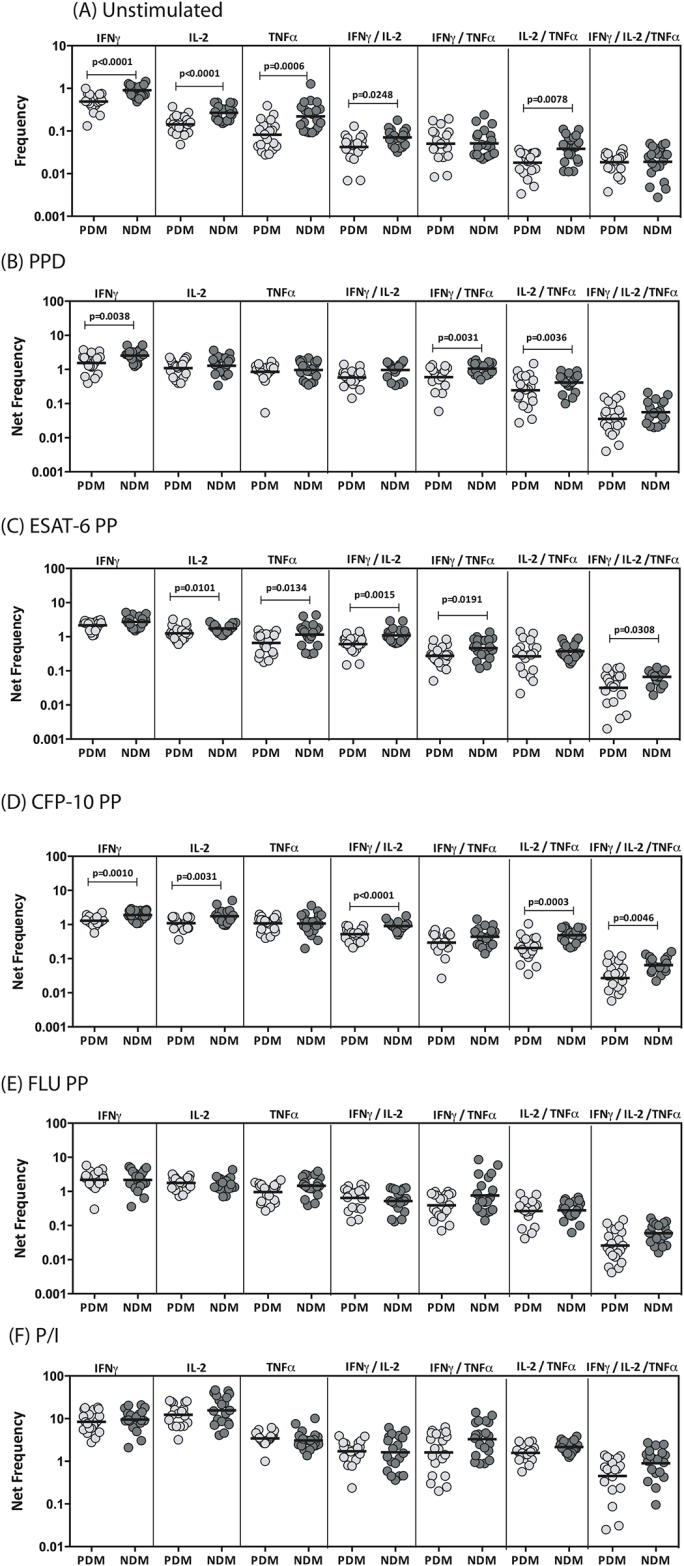
LTB-PDM is associated with decreased spontaneously expressed and antigen - induced frequency of CD4^+^ mono -, dual - and/or multi - functional Th1 cells. Whole blood was cultured with media alone or mycobacterial or control antigens for 6 h and the baseline and antigen—stimulated frequencies of Th1 cells determined. The baseline (A) as well as PPD (B), ESAT-6 peptide pools (C), CFP-10 peptide pools (D), Flu peptide pools (E) and P/I (F) stimulated frequencies of mono–, dual–and multi–functional CD4^+^ Th1 cells in LTB-PDM (n = 20) and LTB-NDM (n = 20) individuals are shown. Each circle represents a single individual and the bars represent the geometric mean values. Net frequencies were calculated by subtracting baseline frequencies from the antigen–induced frequencies for each individual. *P* values were calculated using the Mann-Whitney test.

To determine the influence of PDM on Th1 cells in LTB, we used multi-parameter flow cytometry to define the frequencies CD4^+^ T cells expressing IFNγ, IL-2 and/or TNFα at baseline and following stimulation with either mycobacterial antigens or control antigen or P/I in LTB-PDM and LTB-NDM individuals. As shown in Fig 1A, LTB-PDM individuals exhibited significantly reduced frequencies of mono-functional Th1 (IFNγ or IL-2 or TNFα expressing) or dual-functional Th1 (IFNγ/IL-2 or IL-2/TNFα co-expressing) cells at baseline in comparison to LTB-NDM individuals. Similarly, in response to PPD (Fig 1B), ESAT-6 (Fig 1C) and CFP-10 (Fig 1D), LTB-PDM individuals exhibited significantly decreased frequencies of mono- or dual- functional Th1 cells and in the case of ESAT-6 and CFP-10, multi-functional Th1 (IFNγ/IL-2/TNFα co-expressing) cells as well. In contrast, LTB-PDM individuals did not exhibit any significant difference in the frequencies of mono -, dual—or multi—functional Th1 cells in response to control antigen (Flu peptides) (Fig 1E) or P/I (Fig 1F), indicating that the decreased frequency of Th1 cells present in LTB-PDM individuals was relatively pathogen specific. A representative flow cytometry contour plot showing the baseline, ESAT-6 and P/I stimulated Th1 cytokines is shown in S1 Fig.

### LTB-PDM is associated with decreased frequencies of antigen and mitogen stimulated IL-4^+^ CD4^+^ T cells

**Fig 2:**
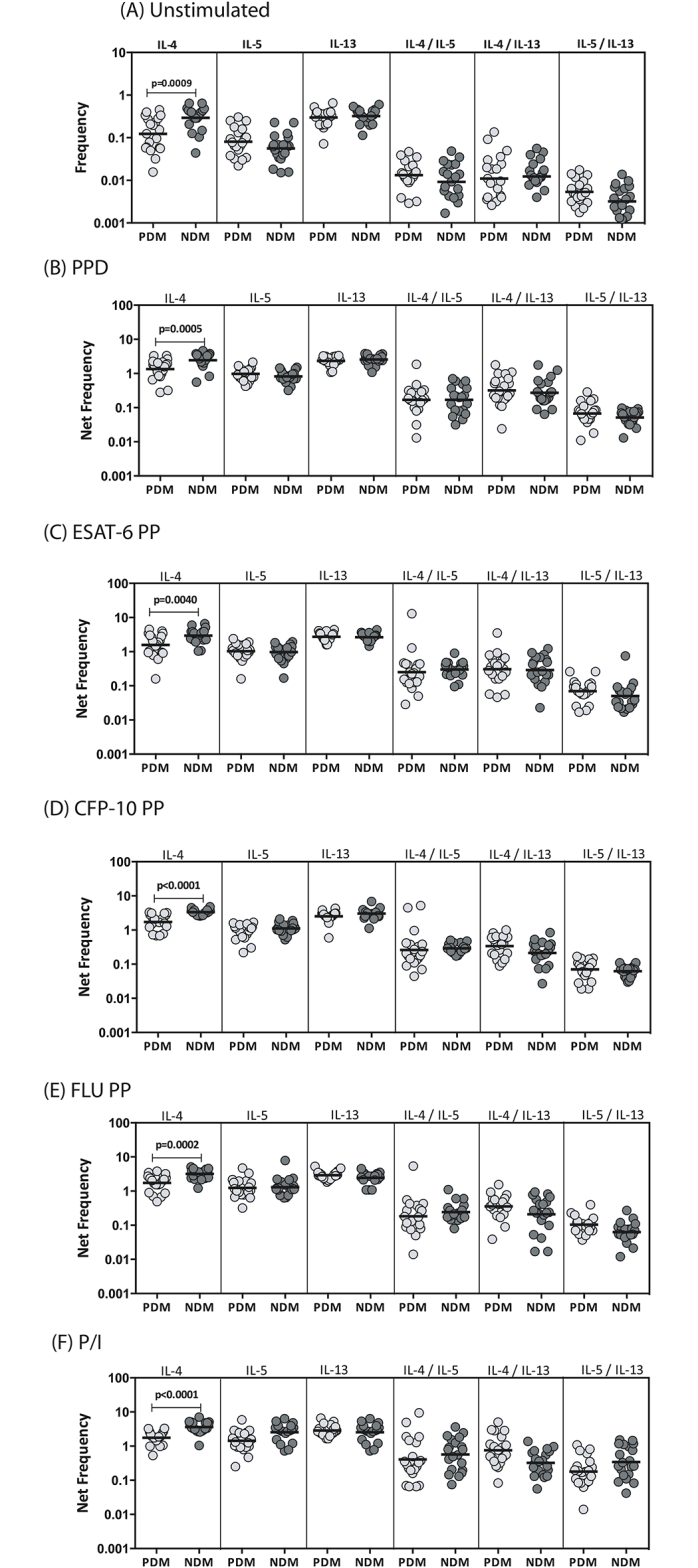
LTB-PDM is associated with decreased spontaneously expressed and antigen - induced frequency of IL-4^+^CD4^+^ Th2 cells. Whole blood was cultured with media alone or mycobacterial or control antigens for 6 h and the baseline and antigen—stimulated frequencies of Th2 cells determined. The baseline (A) as well as PPD (B), ESAT-6 peptide pools (C), CFP-10 peptide pools (D), Flu peptide pools (E) and P/I (F) stimulated frequencies of mono–and dual–functional CD4^+^ Th2 cells in LTB-PDM (n = 20) and LTB-NDM (n = 20) individuals are shown. Each circle represents a single individual and the bars represent the geometric mean values. Net frequencies were calculated by subtracting baseline frequencies from the antigen–induced frequencies for each individual. *P* values were calculated using the Mann-Whitney test.

To determine the influence of PDM on Th2 cells in LTB, we used multi-parameter flow cytometry to define the frequencies CD4^+^ T cells expressing IL-4, IL-5 and/or IL-13 at baseline and following stimulation with either mycobacterial antigens or control antigen or P/I in LTB-PDM and LTB-NDM individuals. As shown in Fig 2A, LTB-PDM individuals exhibited significantly reduced frequencies of IL-4 expressing CD4^+^ T cells at baseline. In addition, in response to PPD (Fig 2B), ESAT-6 (Fig 2C) and CFP-10 (Fig 2D), LTB-PDM individuals exhibited significantly decreased frequencies of IL-4^+^, CD4^+^ T cells in comparison to LTB-NDM individuals. However, this response does not appear to be mycobacterial—antigen specific since LTB-PDM individuals also exhibited significantly decreased frequencies IL-4^+^, CD4^+^ T cells in response to control antigen (Flu peptides) (Fig 2E) or P/I (Fig 2F). A representative flow cytometry contour plot showing the baseline, ESAT-6 and P/I stimulated Th2 cytokines is shown in S1 Fig.

### LTB-PDM is associated with decreased frequencies of antigen-induced mono–and dual–Functional CD4^+^ Th17 cells

**Fig 3:**
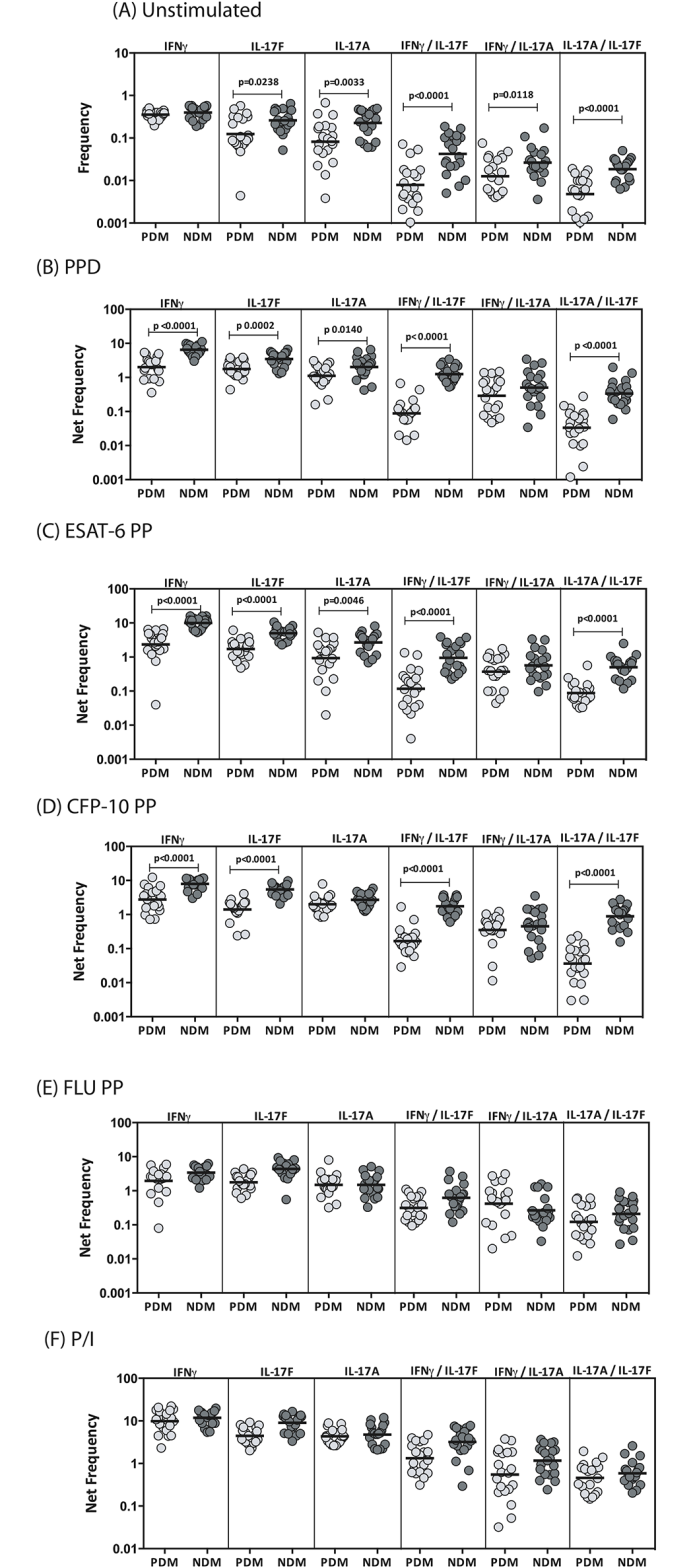
LTB-PDM is associated with decreased spontaneously expressed and antigen - induced frequency of CD4^+^ mono - and/or dual - functional Th17 cells. Whole blood was cultured with media alone or mycobacterial or control antigens for 6 h and the baseline and antigen—stimulated frequencies of Th17 cells determined. The baseline (A) as well as PPD (B), ESAT-6 peptide pools (C), CFP-10 peptide pools (D), Flu peptide pools (E) and P/I (F) stimulated frequencies of mono–and dual–functional CD4^+^ Th17 cells in LTB-PDM (n = 20) and LTB-NDM (n = 20) individuals are shown. Each circle represents a single individual and the bars represent the geometric mean values. Net frequencies were calculated by subtracting baseline frequencies from the antigen–induced frequencies for each individual. *P* values were calculated using the Mann-Whitney test.

To determine the influence of PDM on Th17 cells in LTB, we used multi-parameter flow cytometry to define the frequencies CD4^+^ T cells expressing IL-17A, IL-17F and/or IFNγ at baseline and following stimulation with either mycobacterial antigens or control antigen or P/I in LTB-PDM and LTB-NDM individuals. As shown in Fig 3A, LTB-PDM individuals exhibited significantly reduced frequencies of mono-functional Th17 (IL-17A or IL-17F expressing) or dual-functional Th1 (IFNγ/IL-17F or IFNγ/IL-17A or IL-17F/IL-17A co-expressing) cells at baseline. Similarly, in response to PPD (Fig 3B), ESAT-6 (Fig 3C) and CFP-10 (Fig 3D), LTB-PDM individuals exhibited significantly decreased frequencies of mono- or dual- functional Th17 cells. In contrast, LTB-PDM individuals did not exhibit any significant difference in the frequencies of mono–or dual—functional Th17 cells in response to control antigen (Flu peptides) (Fig 3E) or P/I (Fig 3F), indicating that the decreased frequency of Th17 cells present in LTB-PDM individuals was relatively pathogen specific. A representative flow cytometry contour plot showing the baseline, ESAT-6 and P/I stimulated Th17 cytokines is shown in S1 Fig.

### LTB-PDM is associated with decreased frequencies of antigen-induced mono–and dual–Functional CD8^+^ Tc1 cells

**Fig 4:**
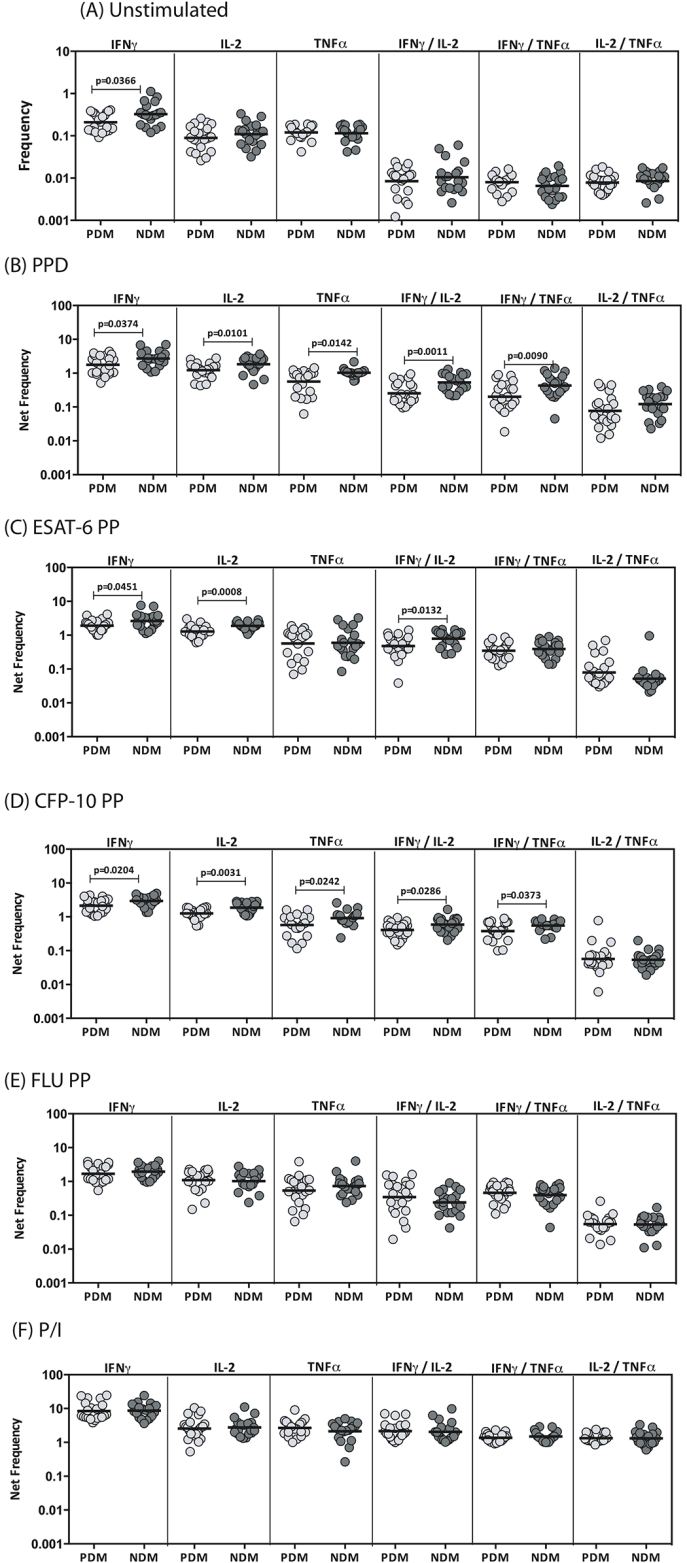
LTB-PDM is associated with decreased spontaneously expressed and antigen - induced frequency of CD8^+^ mono–or dual - functional Tc1 cells. Whole blood was cultured with media alone or mycobacterial or control antigens for 6 h and the baseline and antigen—stimulated frequencies of Tc1 cells determined. The baseline (A) as well as PPD (B), ESAT-6 peptide pools (C), CFP-10 peptide pools (D), Flu peptide pools (E) and P/I (F) stimulated frequencies of mono–and dual–functional CD8^+^ Tc1 cells in LTB-PDM (n = 20) and LTB-NDM (n = 20) individuals are shown. Each circle represents a single individual and the bars represent the geometric mean values. Net frequencies were calculated by subtracting baseline frequencies from the antigen–induced frequencies for each individual. *P* values were calculated using the Mann-Whitney test.

To determine the influence of PDM on Tc1 cells in LTB, we used multi-parameter flow cytometry to define the frequencies CD8^+^ T cells expressing IFNγ, IL-2 and/or TNFαat baseline and following stimulation with either mycobacterial antigens or control antigen or P/I in LTB-PDM and LTB-NDM individuals. As shown in Fig 4A, LTB-PDM individuals exhibited significantly reduced frequencies of mono-functional Tc1 (IFNα) cells at baseline in comparison to LTB-NDM individuals. Similarly, in response to PPD (Fig 4B), ESAT-6 (Fig 4C) and CFP-10 (Fig 4D), LTB-PDM individuals exhibited significantly decreased frequencies of mono- or dual- functional Tc1 cells as well. In contrast, LTB-PDM individuals did not exhibit any significant difference in the frequencies of mono—or dual -functional Tc1 cells in response to control antigen (Flu peptides) (Fig 4E) or P/I (Fig 4F), indicating that the decreased frequency of Tc1 cells present in LTB-PDM individuals was relatively pathogen specific. A representative flow cytometry contour plot showing the baseline, ESAT-6 and P/I stimulated CD8^+^ T cell expression of cytokines is shown in S2 Fig. The gating strategy for CD4^+^ and CD8^+^ T cells is shown in S3 Fig.

### LTB-PDM is associated with decreased frequencies of antigen-induced CD8^+^ Tc2 and Tc17 cells

**Fig 5:**
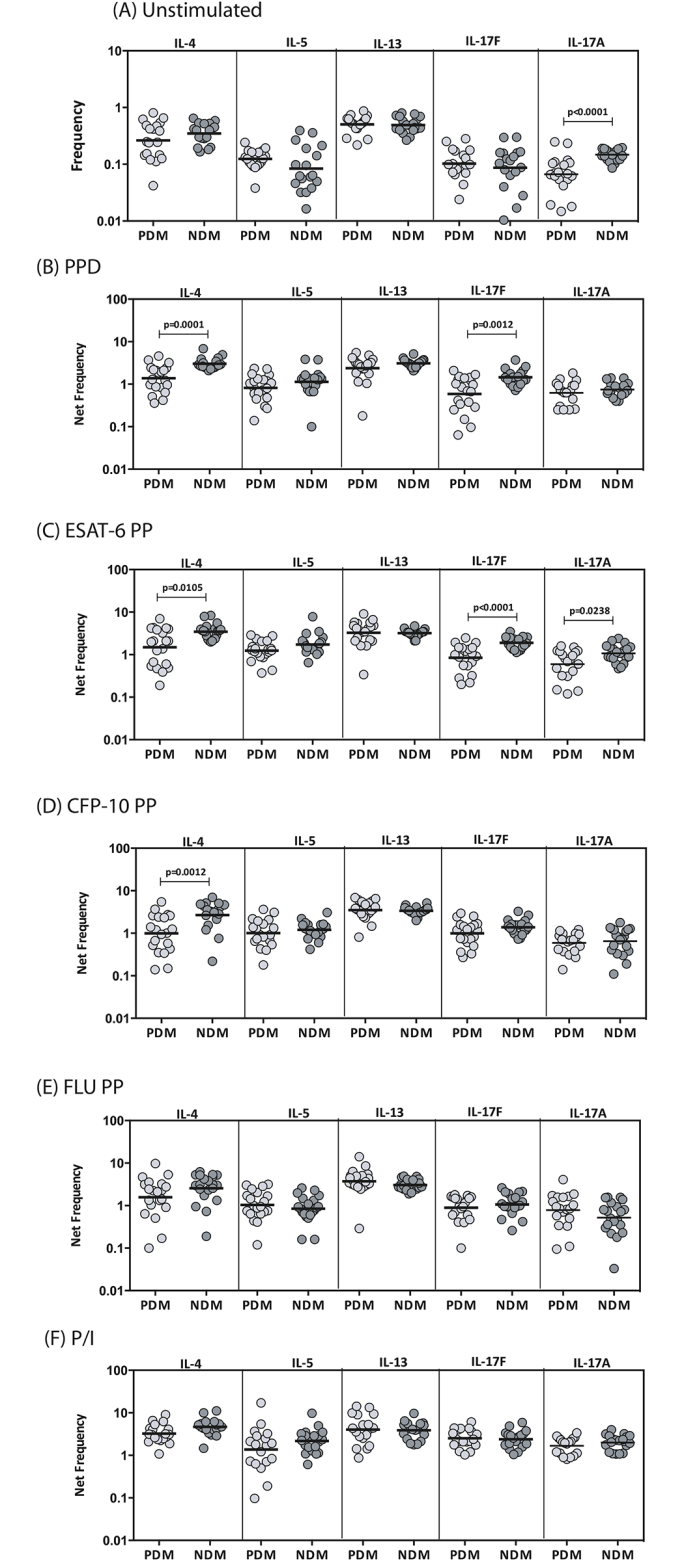
LTB-PDM is associated with decreased spontaneously expressed and antigen - induced frequency of CD8^+^ Tc2 and Tc17 cells. Whole blood was cultured with media alone or mycobacterial or control antigens for 6 h and the baseline and antigen—stimulated frequencies of Tc2 and Tc17 cells determined. The baseline (A) as well as PPD (B), ESAT-6 peptide pools (C), CFP-10 peptide pools (D), Flu peptide pools (E) and P/I (F) stimulated frequencies of CD8^+^ Tc2 and Tc17 cells in LTB-PDM (n = 20) and LTB-NDM (n = 20) individuals are shown. Each circle represents a single individual and the bars represent the geometric mean values. Net frequencies were calculated by subtracting baseline frequencies from the antigen–induced frequencies for each individual. *P* values were calculated using the Mann-Whitney test.

To determine the influence of PDM on Tc2 and Tc17 cells in LTB, we used multi-parameter flow cytometry to define the frequencies CD8^+^ T cells expressing IL-4, IL-5, IL-13, IL-17A or IL-17F at baseline and following stimulation with either mycobacterial antigens or control antigen or P/I in LTB-PDM and LTB-NDM individuals. As shown in Fig 5A, LTB-PDM individuals exhibited significantly reduced frequencies of mono-functional Tc17 (IL-17A) cells at baseline in comparison to LTB-NDM individuals. Similarly, in response to PPD (Fig 5B), ESAT-6 (Fig 5C) and CFP-10 (Fig 5D), LTB-PDM individuals exhibited significantly decreased frequencies of Tc2 and Tc17 cells as well. In contrast, LTB-PDM individuals did not exhibit any significant difference in the frequencies of Tc2 or Tc17 cells in response to control antigen (Flu peptides) (Fig 1E) or P/I (Fig 1F), indicating that the decreased frequency of Tc2 and Tc17 cells present in LTB-PDM individuals was relatively pathogen specific.

## Discussion

While the association between DM and the risk of active TB has been well documented [6], the nature of the relationship between PDM and latent TB is not well understood. We have previously shown that DM is associated with the down modulation of CD4^+^ Th1, Th2 and Th17 responses in LTB, a response mediated in part by IL-10 and TGFβ [18]. Similarly, LTB-DM co-morbidity is also characterized by down regulated cytokine expression in mycobacterial antigen–induced CD8^+^ T cells [19]. In addition, both PDM and DM are known to be associated with diminished levels of circulating and antigen–induced Type 1, Type 17 and other pro-inflammatory cytokines in LTB individuals [20]. Moreover, recent clinical studies delineate an important association of PDM with both increased risk of latent TB infection and increased risk of active TB disease [7,8]. Thus, the elucidation of the immunological interaction between PDM and LTB or active TB becomes of paramount importance.

CD4^+^ T cells play a pivotal role in determining the clinical outcome following exposure to *M*. *tuberculosis* [11]. Thus, CD4^+^ T cells expressing Th1 pattern of cytokines are thought to be crucial players in the immune response to the TB bacillus [11,12]. In addition, CD4^+^ T cells typically producing more than one cytokine are thought to be associated with a protective immune response and indeed, these multi-functional Th1 cells have been shown to be enhanced in latent infection compared to active disease [15,16]. Moreover, these CD4^+^ T cells have also been shown to be inversely associated with bacterial load, again implying a role for these cells as correlates of protective immunity [15]. Our findings demonstrate that both at baseline and following stimulation with mycobacterial antigens, the frequencies of mono—and dual—functional Th1 cells (and in the case of ESAT-6 and CFP-10 stimulation, multi–functional Th1 cells) is decreased in PDM individuals compared to NDM individuals with LTB. Furthermore, our data clearly demonstrate the relatively pathogen or antigen–specific nature of this down modulation since the diminution in the frequencies of mono–and dual–functional Th1 cells in LTB-PDM individuals is almost completely abolished when stimulation with a control antigen or polyclonal stimulus was used. Our study, therefore, highlight the association of diminished Th1 responses in LTB-PDM co-morbidity.

In addition to CD4^+^ Th1 cells, Th17 cells are also thought to be important players in mediating cellular immunity to TB [12]. These cells, defined by the production of IL-17A and IL-17F, are major players in protection against TB infection and are essential for protective immunity against hyper-virulent TB strains [21]. Our study also reveals an important alteration in the baseline as well as the mycobacterial antigen–induced frequencies of CD4^+^ Th17 cells. Our data shows that mono—and dual—functional Th17 cells are also present at decreased frequencies in LTB-PDM in comparison to LTB-NDM. Therefore, a diminished Th17 response, similar to the Th1 response, occurs in LTB individuals with prediabetes. Similar to our data on Th1 and Th17 cells, our data also reveal an important association of decreased frequencies of IL-4^+^, CD4^+^ T cells with PDM. Of interest, PDM was only associated with alterations in the IL-4^+^ T cell compartment with no modulation in the frequencies of other Th2 cells, the relevance of which needs to be further explored. Nevertheless, this finding suggests that the diminution in the frequencies of Th1 and Th17 cells cannot simply be attributed to increased frequencies of Th2 cells and the resultant cross-regulation of CD4^+^ T cell responses.

Recent data clearly highlight an important role for CD8^+^ T cells in immunity to TB [17,22]. In addition, alterations in CD8^+^ T cell cytokine expression is an important hallmark of diabetes–active TB and diabetes–latent TB co-morbidities [19,23]. To our knowledge, this is the first investigation into the role of CD8^+^ T cells in prediabetes with latent TB. Our data reveal that similar to CD4^+^ Th1 cells, CD8^+^ Tc1 cells are also present at diminished frequencies in response to mycobacterial antigen stimulation in LTB-PDM individuals. This is accompanied by decreased frequencies of IL-4^+^ Tc2 cells and IL-17A or IL-17F expressing Tc17 cells. Hence, the effect of PDM on the adaptive immune responses to TB clearly extends to the CD8^+^ T cell compartment as well.

Our study suffers from several limitations. Being a cross-sectional study, we are unable to attribute cause and effect relationships and in addition, the sample size is quite moderate. Moreover, the mechanism behind the down modulation of T cell cytokine responses has not been explored in this study. Moreover, other concomitant infections, co-morbidities or nutritional deficiencies could also impact the findings. Also, this study could not determine the temporal connection between prediabetes and LTB as it is not possible to ascertain which came first. Thus, the results of this study need to be further corroborated in a larger study with follow up samples. However, our data clearly illustrate the antigen–specificity of this immune down modulation for Th1 and Th17 cells. While the mechanism underlying this down modulation needs to be explored further, there are no similar studies on PDM individuals with other co-existent infections and hence this to our knowledge is the first to highlight this immunological nexus.

Our data clearly reveal an important association of prediabetes with modified immune responses (both CD4^+^ and CD8^+^ T cell) in latent TB infection. They suggest that down modulation of T cell cytokine responses could be an important feature in LTB-DM and could potentially contribute to the increased risk posed by prediabetes in the pathogenesis of active TB.

## Supporting information

S1 Fig Representative flow cytometry plot for cytokine expression from CD4^+^ T cells. Whole blood was cultured with media alone or mycobacterial antigens or PMA/ Ionomycin for 6 h and the baseline and antigen—specific frequencies of Th1 cells determined. A representative whole-blood intracellular cytokine assay flow data from a LTB-PDM individual showing expression of Th1, Th2 and Th17 cytokines at baseline and following stimulation with ESAT-6 peptide pools or PMA/Ionomycin. The plots shown are gated on CD3^+^CD4^+^ T cells.
(PDF)

S2 Fig Representative flow cytometry plot for cytokine expression from CD8^+^ T cells. Whole blood was cultured with media alone or mycobacterial antigens or PMA/ Ionomycin for 6 h and the baseline and antigen—specific frequencies of Th1 cells determined. A representative whole-blood intracellular cytokine assay flow data from a LTB-PDM individual showing expression of Th1, Th2 and Th17 cytokines at baseline and following stimulation with ESAT-6 peptide pools or PMA/Ionomycin. The plots shown are gated on CD3^+^CD8^+^ T cells.
(PDF)

S3 Fig Gating strategy for CD4^+^ and CD8^+^ T cells. The gating strategy for CD4^+^ and CD8^+^ T cells from the same representative PDM individuals is shown.
(PDF)

S1 Table Excel sheet with all the raw data from the study. The raw data values of the frequencies of CD4^+^ and CD8^+^ T cells expressing Th1/Tc1, Th2/Tc2 and Th17/Tc17 cytokines is given in the excel sheet.
(XLS)

## Abbreviations

DM: diabetes mellitus
LTB: Latent tuberculosis
NDM: no diabetes
PDM: prediabetes
P/I: PMA-Ionomycin
Tc: cytotoxic T cell
Th: helper T cell
