# Supplementary figures and images for: Prediabetes is associated with the modulation of antigen-specific Th1/Tc1 and Th17/Tc17 responses in latent Mycobacterium tuberculosis infection
Source: PLoS One. 2017 May 30;12(5):e0178000. doi: 10.1371/journal.pone.0178000 (PMC5448753; doi:10.1371/journal.pone.0178000)

S Fig 1

Unstimulated

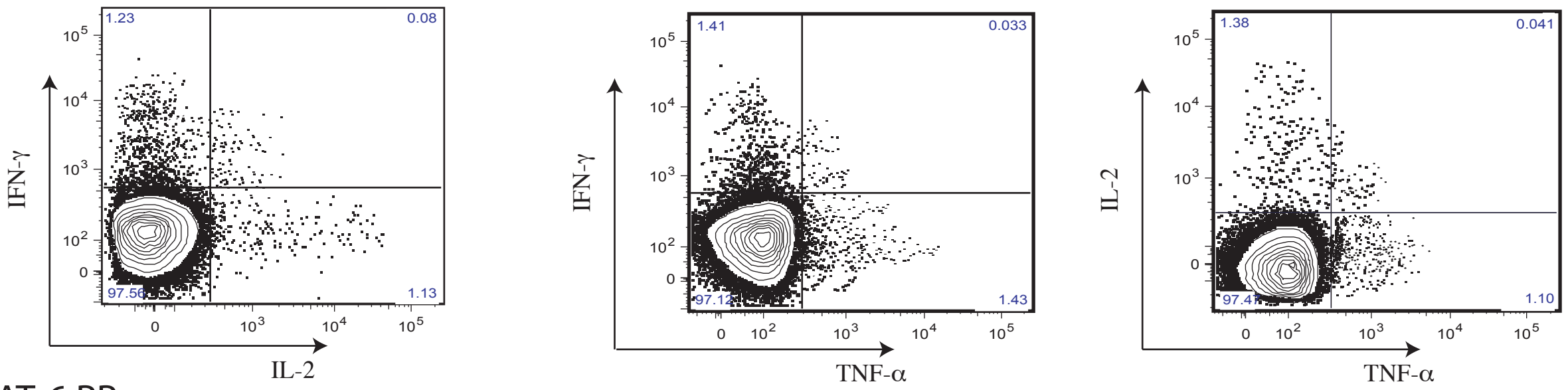

Unstimulated

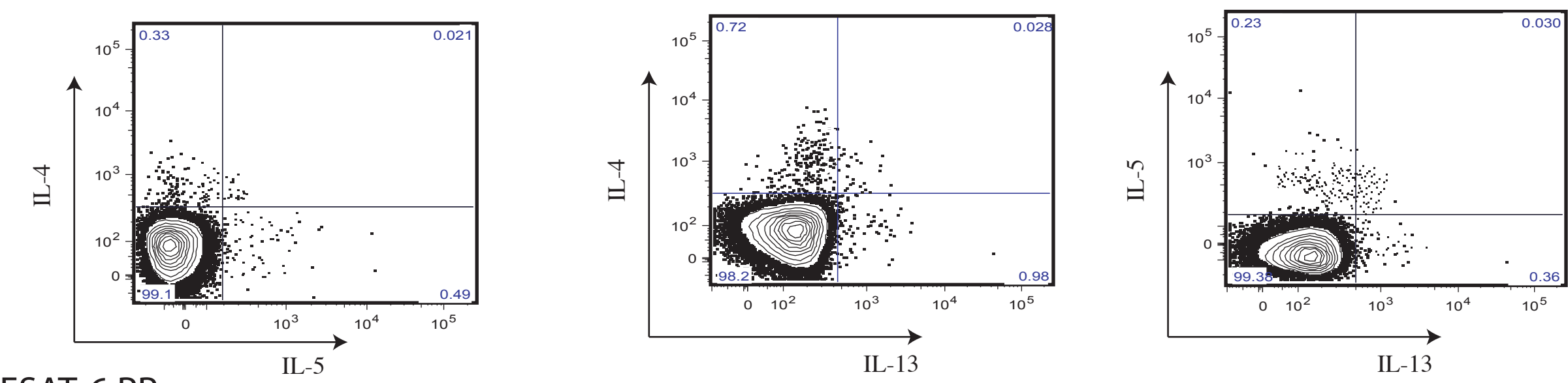

Unstimulated

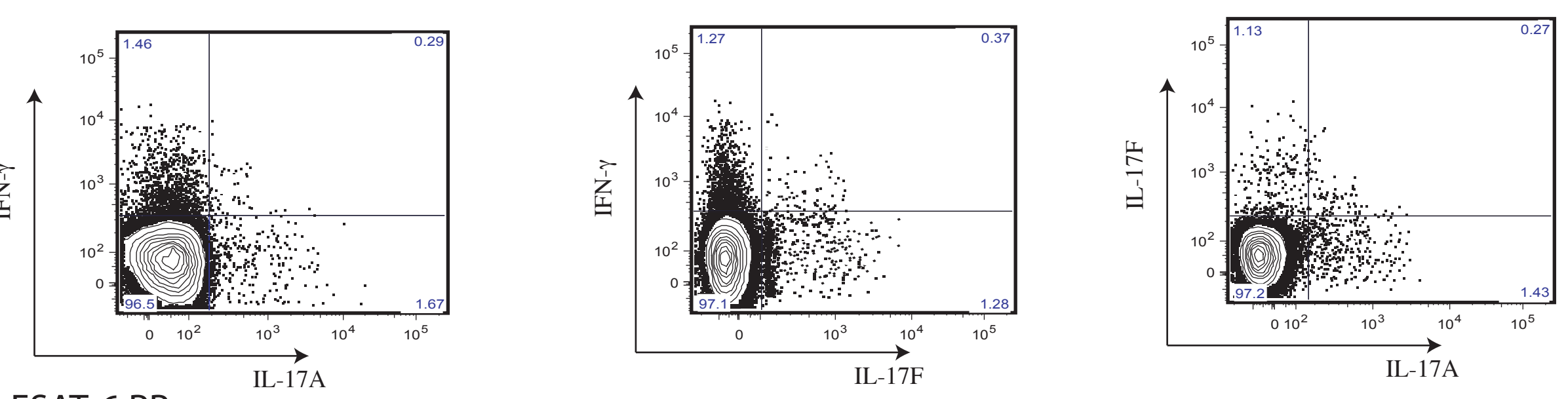

ESAT-6 PP

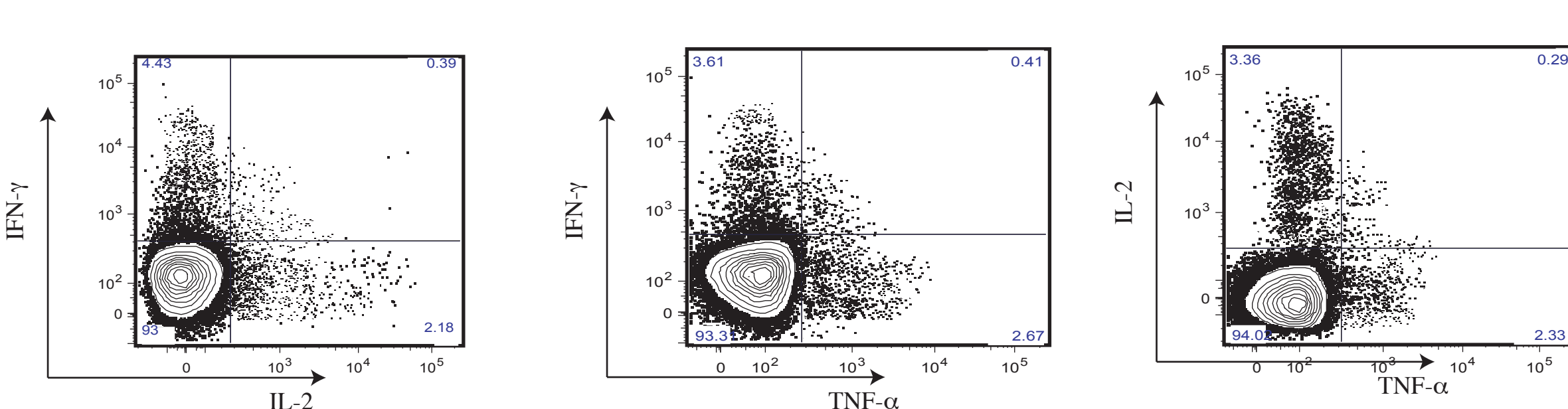

ESAT-6 PP

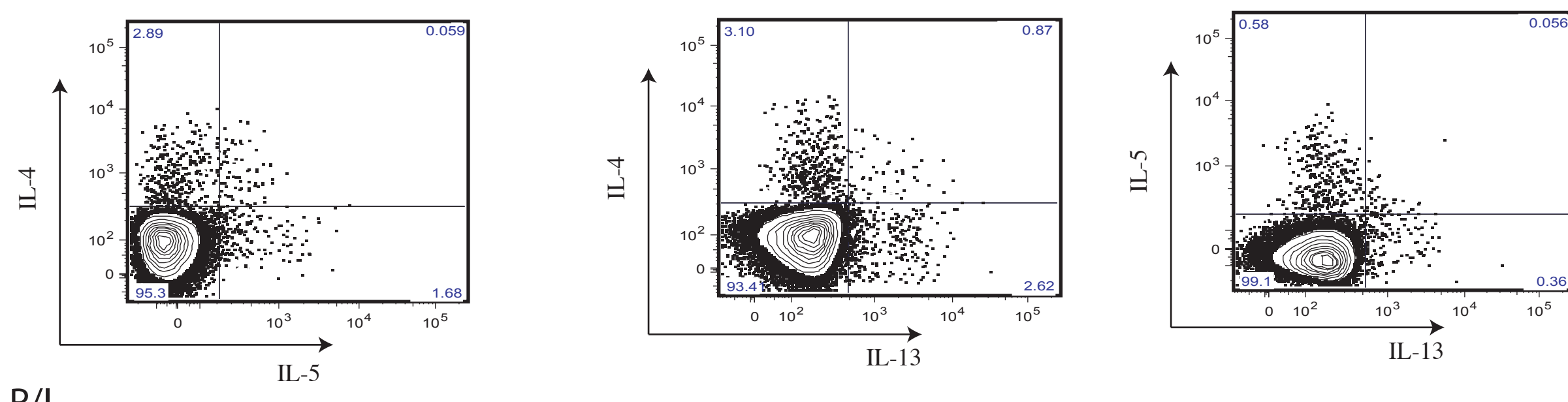

ESAT-6 PP

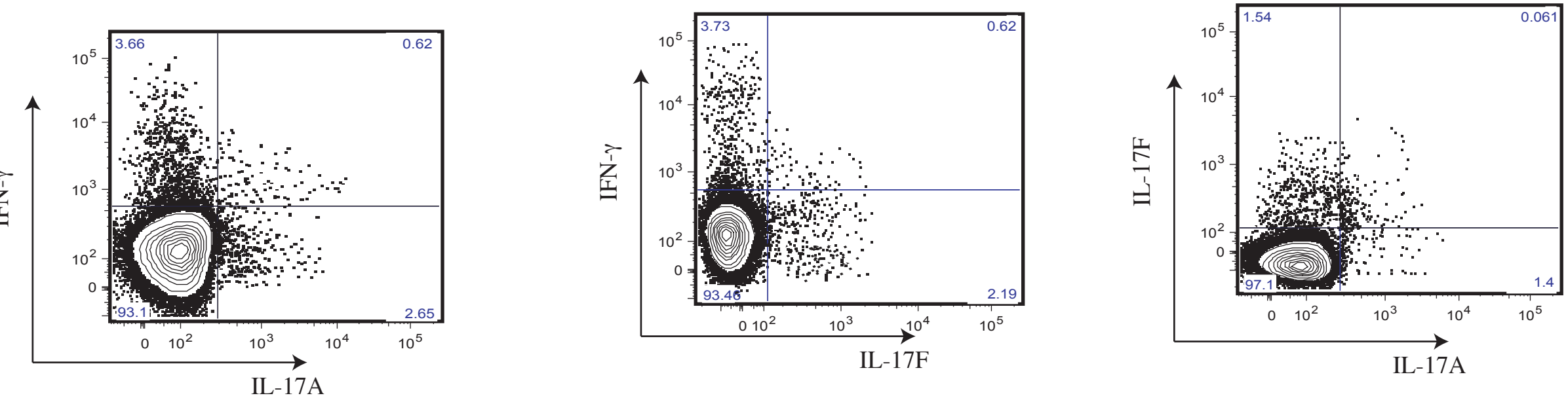

P/I

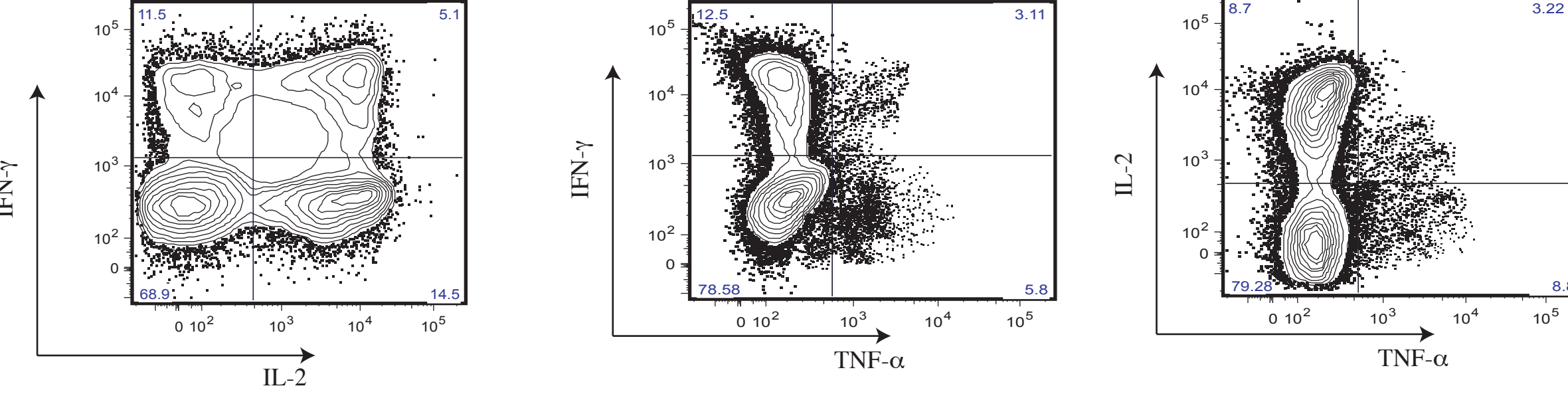

P/I

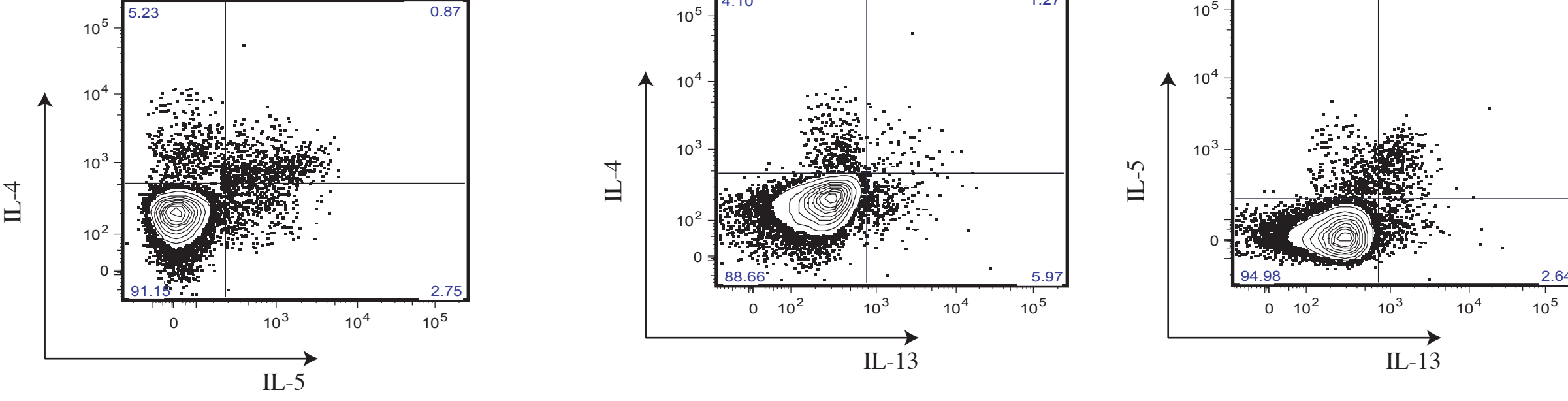

P/I

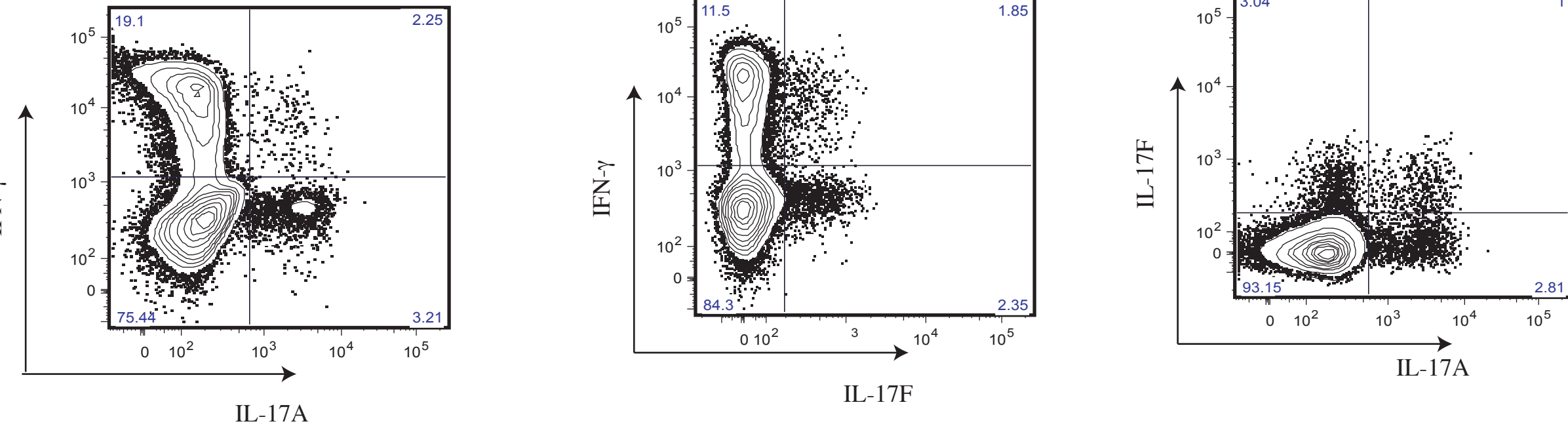

Supplement: S1 Fig — Whole blood was cultured with media alone or mycobacterial antigens or PMA/ Ionomycin for 6 h and the baseline and antigen—specific frequencies of Th1 cells determined. A representative whole-blood intracellular cytokine assay flow data from a LTB-PDM individual showing expression of Th1, Th2 and Th17 cytokines at baseline and following stimulation with ESAT-6 peptide pools or PMA/Ionomycin. The plots shown are gated on CD3+CD4+ T cells. (PDF) [file pone.0178000.s001.pdf]

S Fig 2

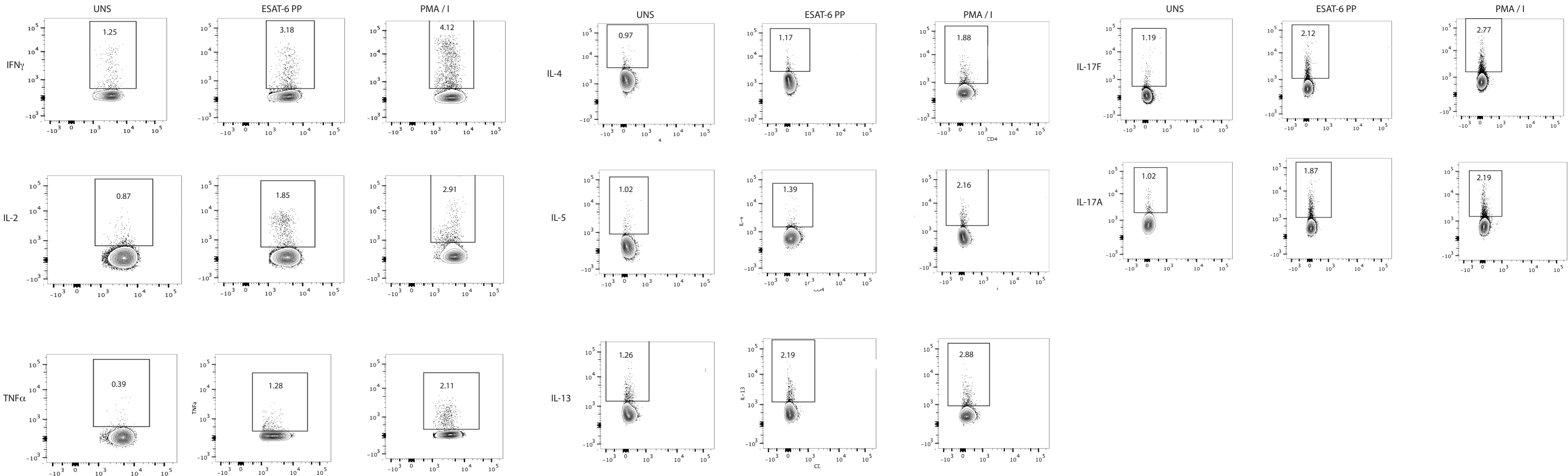

Supplement: S2 Fig — Whole blood was cultured with media alone or mycobacterial antigens or PMA/ Ionomycin for 6 h and the baseline and antigen—specific frequencies of Th1 cells determined. A representative whole-blood intracellular cytokine assay flow data from a LTB-PDM individual showing expression of Th1, Th2 and Th17 cytokines at baseline and following stimulation with ESAT-6 peptide pools or PMA/Ionomycin. The plots shown are gated on CD3+CD8+ T cells. (PDF) [file pone.0178000.s002.pdf]

S Fig 3

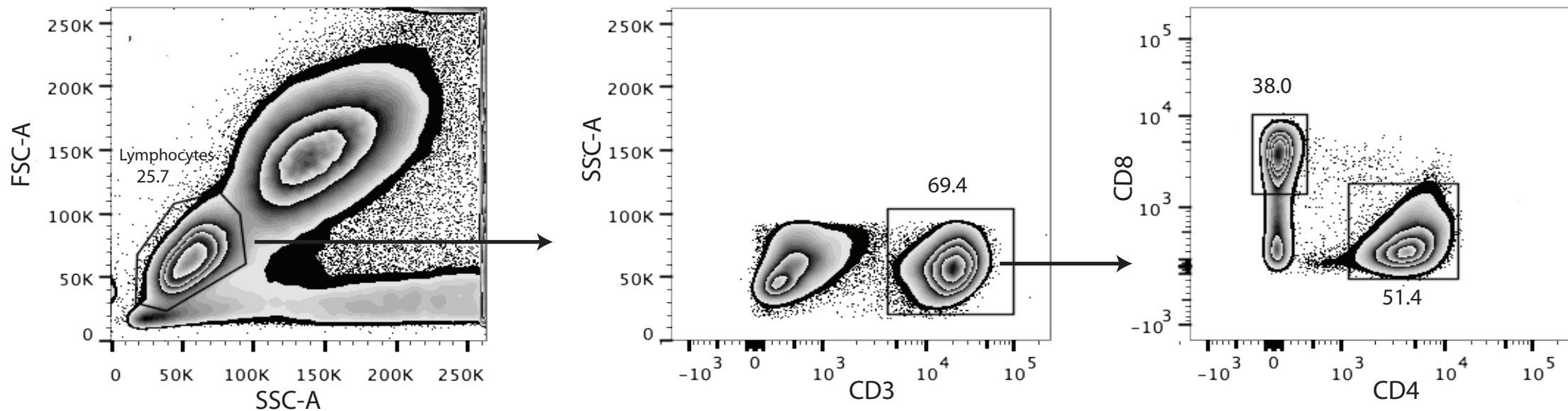

Supplement: S3 Fig — The gating strategy for CD4+ and CD8+ T cells from the same representative PDM individuals is shown. (PDF) [file pone.0178000.s003.pdf]
